# Retnla Overexpression Attenuates Allergic Inflammation of the Airway

**DOI:** 10.1371/journal.pone.0112666

**Published:** 2014-11-21

**Authors:** Mi-Ran Lee, Dahee Shim, Jihye Yoon, Hyung Seok Jang, Se-Woong Oh, Suk Hyo Suh, Jae-Hoon Choi, Goo Taeg Oh

**Affiliations:** 1 Department of Life Sciences, Ewha Womans University, Seoul, Republic of Korea; 2 Department of Life Science, College of Natural Sciences, Research Institute for Natural Sciences, Hanyang University, Seoul, Republic of Korea; 3 Yuhan Research Institute, Yuhan Corporation, Gongse-Dong, Giheung-Gu, Yongin-Si, Gyeonggi-Do, Republic of Korea; 4 Department of Physiology Medical School, Ewha Womans University, Seoul, Republic of Korea; Yale University, United States of America

## Abstract

Resistin-like molecule alpha (Retnla), also known as ‘Found in inflammatory zone 1’, is a secreted protein that has been found in bronchoalveolar lavage (BAL) fluid of ovalbumin (OVA)-induced asthmatic mice and plays a role as a regulator of T helper (Th)2-driven inflammation. However, the role of Retnla in the progress of Th2-driven airway inflammation is not yet clear. To better understand the function of Retnla in Th2-driven airway inflammation, we generated Retnla-overexpressing (*Retnla*-Tg) mice. *Retnla*-Tg mice showed increased expression of Retnla protein in BAL fluid and airway epithelial cells. Retnla overexpression itself did not induce any alteration in lung histology or lung function compared to non-Tg controls. However, OVA-sensitized/challenged *Retnla*-Tg mice had decreased numbers of cells in BAL and inflammatory cells accumulating in the lung. They also showed a reduction in mucus production in the airway epithelium, concomitant with a decreased Muc5ac level. These results were accompanied by reduced levels of Th2 cytokines, including interleukin (IL)-4, IL-5, and IL-13, with no effect on levels of OVA-specific immunoglobulin isotypes. Furthermore, phosphorylation of ERK was markedly reduced in the lungs of OVA-challenged *Retnla*-Tg mice. Taken together, these results indicates that Retnla protects against Th2-mediated inflammation in an experimental mouse model of asthma, suggesting that therapeutic approaches to enhance the production of Retnla or Retnla-like molecules could be valuable for preventing allergic lung inflammation.

## Introduction

Allergic asthma is a chronic inflammatory disorder of the airway, driven by an imbalance in T helper (Th) cell responses, leading to chronic airway inflammation, fibrosis in the lamina reticularis and adventitia of the airway, and mucus hypersecretion in the lung [1]. Overactive Th2 responses drive the development of asthma by triggering the secretion of cytokines, in particular, interleukin (IL)-4, IL-5, and IL-13, which are thought to play central roles in the initiation and maintenance of allergic responses by contributing to asthmatic features, including Th2 differentiation, lung eosinophilia, mucus hypersecretion, mast cell hyperplasia, and bronchial hyperresponsiveness [1], [2].

Resistin-like molecule alpha (Retnla), also known as ‘Found in inflammatory zone 1’ or ‘Hypoxia-induced mitogenic factor’, belongs to a family of small cysteine-rich secreted proteins, including Retnla/Relm-α/FIZZ1, Retnlb/Relm-β/FIZZ2, Resistin/FIZZ3 and Retnlg/Relm-γ/FIZZ4 [3], [4]. It was initially found in bronchoalveolar lavage (BAL) fluid from mice experiencing ovalbumin (OVA)-induced allergic airway inflammation. Interestingly, Retnla expression is highly induced during the Th2 cytokine-dependent inflammatory response and therefore is an indicator of the alternative activation of macrophages [5]–[7]. Function studies have shown the various role of Retnla in the pathogenesis of several inflammatory diseases, including parasitic diseases, asthma, and colitis [3], [8], [9]. However, the results of several previous papers regarding the function of Retnla in lung Th2-mediated inflammation are currently controversial. For example, Retnla has been suggested to enhance wound-repair and fibrosis at sites of Th2-mediated inflammation [10]–[13]. More recently, it has been demonstrated that Retnla deficiency exacerbated lung inflammation induced by parasitic infection via enhancement of Th2 immune responses, suggesting that Retnla is a negative regulator of Th2-mediated lung inflammation [14], [15]. Consistent with the effect of Retnla in regulating Th2 immunity, an *in vitro* study showed that recombinant Retnla antagonizes the effects of nerve growth factor, which can exacerbate allergic pulmonary inflammation [3]. Counter to this, recent work using *Retnla*-deficient mice showed that Retnla is dispensable for Th2-associated allergen-driven lung inflammation [16], and another study reported that recombinant Retnla increased proliferation and growth factor expression in pulmonary endothelial cells [17], [18]. Moreover, recombinant Retnla increased carbachol-induced tracheal smooth muscle contraction through activation of mitogen-activated protein kinase (MAPK) signaling [19]. The direct administration of recombinant Retnla to mice promoted airway eosinophilia, fibrosis, and epithelial thickening [20]. Although several previous papers have reported on the involvement of Retnla in airway Th2-mediated inflammatory processes, the results are heterogeneous, and the exact function of Retnla in allergic lung inflammation is not completely understood.

To delineate the functions of Retnla in allergen-induced pulmonary inflammation, we analyzed allergic lung inflammation in *Retnla*-overexpressing (*Retnla*-Tg) mice. Our data indicates that *Retnla*-Tg mice did not have affected lung function or pulmonary fibrosis, but they were more resistant to lung inflammation, suggesting that Retnla plays an anti-inflammatory role in allergic lung inflammation.

## Materials and Methods

### Generation of Retnla transgenic mice

*Retnla*-Tg mice were generated as previously described [21]. Two founders (lines 1800 and 0011, C57BL/6J background) were identified by PCR analysis of tail DNA using the following primer set: forward, 5′-CTCCACTGTAACGAAGACTC-3′ and reverse, 5′-GCAGTGGTCCAGTCAACGA-3′. The transgenic (Tg) mice were bred to be hemizygous for the transgene. Non-Tg littermates were used as controls. Since the expression levels of Retnla did not differ significantly between the two Tg lines (Figure S1), the *Retnla* line 1800 was mainly used for this study. Mice were housed in a room with a 12 h light cycle and were provided a standard diet *ad libitum* and had unrestricted access to drinking water. The study was conducted in accordance with criteria outlined in the Guide for the Care and Use of Laboratory Animals prepared by the Animal Care Committee of Ewha Womans University.

### Northern blotting

Total tissue RNA was extracted using Isol-RNA Lysis reagent (5 PRIME, Gaithersburg, MD, USA) according to the manufacturer's protocol. For Northern blot analysis, 10 µg of total RNA was separated by electrophoresis on a 1% denaturing agarose gel and transferred to a nylon membrane, to which it was cross-linked by UV irradiation. A cDNA fragment of mouse Retnla was radiolabeled and used as a probe. The membrane was hybridized at 68°C for 1 h in ExpressHyb hybridization solution (Clontech, Mountain View, CA, USA) with a ^32^P-labeled probe. The membrane was washed twice at 50°C for 40 min in 0.1×SSC/0.1% SDS.

### Respiratory function

Respiratory function in mice was measured as described previously [22]. Briefly, *in vivo* respiratory function, including respiratory frequency (fR; number of breaths per min, BPM), minute ventilation (V_E_; ml/min), tidal volume (V_T_; ml), peak expiratory flow (PEF), and peak inspiratory flow (PIF), was measured using whole-body plethysmography (model PLY 3211; Buxco Electronics, Sharon, CT, USA) under unrestrained conscious conditions. *In vivo* airway responsiveness was also measured in *Retnla-*Tg and non-Tg mice after stimulation with increasing doses of inhaled methacholine using whole-body plethysmography under unrestrained conscious conditions.

### Allergen-induced airway inflammation

Asthma models were induced by sensitizing mice to chicken egg ovalbumin (OVA), followed by aerosol or intranasal challenges as previously described [23], with minor modifications. Briefly, the mice were sensitized primarily by intraperitoneal injection with 200 µg OVA (Sigma, St Louis, MO, USA) and 2.5 mg aluminum hydroxide (alum; Pierce, Rockford, IL, USA) as an adjuvant, followed by a second intraperitoneal injection of 20 µg OVA and 2.5 mg alum administered 10 days later. After an additional 10 days, mice were challenged with an aerosol of 1% OVA using a nebulizer for 30 min daily on three consecutive days (Beurer, Ulm, Baden-Württemberg, Germany). Three days after the final challenge, the mice were anesthetized, bronchoalveolar lavage fluid was harvested, and the lungs were excised for histological analysis.

### Bronchoalveolar lavage

BAL was performed as previously described [24], with minor modifications. Briefly, the mice were anesthetized, and BAL fluid was collected by intratracheal insertion of a catheter and lavage with two 1 ml aliquots of saline. Inflammatory cells in the BAL fluids were collected by centrifugation at 1,000 g for 10 min, and the supernatant was concentrated to approximately 200 µl using a Centricon spin column (MW cut-off 3,000-daltons; Millipore, Billerica, MA, USA) for immunoblotting. The BAL cells were washed once in PBS and resuspended in 100 µl of PBS. The total number of cells per milliliter of BAL fluid was determined with the use of a hemocytometer (Hausser-Scientific, Horsham, PA, USA).

### Flow cytometric analysis

The lung cells were isolated by digestion with 400 U/ml collagenase D in PBS containing Ca^2+^ Mg^2+^ at 37°C for 25 min, followed by filtration through a 70-µm cell strainer. The lung cells or BAL cells were resuspended in PBS containing 2% FBS. Nonspecific binding was blocked by incubating with 12.5 µg/ml Fc Block (93, eBioscience) for 15 min at 4°C. Samples were incubated with anti-mouse antibodies including FITC-conjugated CD11b (M1/70, BioLegend), CD3 (17A2, BioLegend), PE-conjugated CD64 (X54-5/7.1, BioLegend), NK1.1 (PK136, BioLegend), PerCP-conjugated CD45 (30-F11, BioLegend), PE/Cy-7-conjugated CD11c (N418, BioLegend), APC-conjugated MHCII (M5/114.15.2, BioLegend), Ly6G (1A8, BioLegend), APC/Cy-7-conjugated CD19 (6D5, BioLegend), CD11b (M1/70, BD Pharmingen), and Pacific Blue-conjugated Siglec-F (E50-2440, BD Pharmingen) for 30 min at 4°C. Samples were washed twice with FACS buffer, and flow cytometry was performed after gating on the CD45^+^ leukocyte population using a BD Canto II flow cytometer (BD biosciences). Results were analyzed with FlowJo software ver. 9.3.2.

### Histopathology and fibrosis

After fixation for 24 h in 10% neutral buffered formalin, lung tissues were routinely processed, embedded in paraffin, and cut into 5-µm sections [25], [26]. For immunostaining of Retnla, after blocking non-specific binding with a blocking reagent (CAS-block; Zymed, San Francisco, CA, USA), the slides were incubated at 4°C overnight with rabbit anti-mouse Retnla polyclonal antibody at a 1:200 dilution. After washing, a 1:200 dilution of secondary goat anti-rabbit biotinylated IgG antibody (Vector labs, Burlingame, CA, USA) was applied for 1 h at room temperature. Biotinylated antibody was detected using Texas-red conjugated avidin (Vector Labs, Burlingame, CA, USA) under a fluorescence microscope. Negative control tissues were prepared in the same manner described above, except for the omission of the primary antibody and the substitution of non-immunized normal rabbit serum. The inflammation score was determined by double-blind histological analysis with the following criteria: 0 for no infiltration; 1 for ∼one layer; 2 for ∼three layers; 3 for ∼five layers; 4 for ∼seven layers; or 5 for more than seven layers of inflammatory cells around bronchioles, peribronchial arteries, and veins. Quantification of Alcian blue-positive epithelial cells was performed by manual assessment within randomly chosen sections (five sections analyzed/animal, per 200× field). Lung hydroxyproline content was determined using a hydroxyproline colorimetric assay kit according to the manufacturer's instructions (Biovision, Milpitas, CA).

### Quantitative real-time RT-PCR analysis

Peritoneal macrophages were isolated from 8-week-old *Retnla*-Tg and non-Tg male mice by peritoneal lavage with 10 ml of ice-cold PBS and were plated in RPMI-1640 medium (Invitrogen) containing 10% heat-inactivated FBS (Hyclone). Total RNA from cells and tissues was extracted using Trizol (5 PRIME, Gaithersburg, MD, USA) and quantified with a spectrophotometer; 1.5 µg of RNA was then reverse transcribed into complementary DNA using the RevertAid First Strand cDNA Synthesis Kit (Fermentas, Pittsburgh, PA, USA) according to the manufacturer's instructions. qPCR reactions were performed using SYBR Green PCR master mix (KAPA Biosystems, Wilmington, MA, USA). The following primers were used: Retnla forward, 5′-CTCCACTGTAACGAAGACTC-3′ and reverse, 5′-GCAGTGGTCCAGTCAACGA-3′; Il-4 forward, 5′-CTTCCAAGGTGCTTCGCATA-3′; Il-4 reverse, 5′-CTTATCGATGAATCCAGGCAT-3′; Il-5 forward, 5′-GACGAGGCAGT TCCTGGAT-3′; Il-5 reverse, 5′-GCATATGGTATCCCTTGCATT-3′; Il-13 forward, 5′-GAGGATATTGCATGGCCTCT-3′; Il-13 reverse, 5′-GTTGCTTTGTGTAGCTGAGCA-3′; Muc5ac forward, 5′-TGTGTCTGTACCTACAACGG-3′; Muc5ac reverse, 5′-AGGGCTCTT CACAGACAATA-3′; *β*-actin forward, 5′-ACGGCCAGGTCATCACTATTG-3′; *β*-actin reverse, 5′ CACAGGATTCCATACCCAAGAAG′ [27]. The relative amount of mRNA was determined using the comparative threshold (Ct) method. Ct values for the target gene were normalized against those of *β*-actin. Data are expressed as the fold-change relative to the control sample.

### ELISA

The concentrations of cytokines and immunoglobulin isotypes in BAL supernatant were determined using a commercially available ELISA kit according to the manufacturer's protocol. The species identified were IL-4, IL-5, IL-10, and IL-13 (R&D Systems, Minneapolis, MN); IgG1, IgG2a, and IgE (Abcam, Cambridge, MA) and IgG2c (eBioscience, San Diego, CA, USA).

### Immunoblotting

The protein from lung tissues was extracted with RIPA buffer (25 mM Tris-HCl, pH 7.6, 150 mM NaCl, 1% NP-40, 1% sodium deoxycholate, 0.1% SDS) containing complete protease inhibitor cocktail (Roche). Forty micrograms of lung extract or 5 µl of BAL fluid concentrated 10-fold by Centricon (Millipore, Billerica, MA, USA) were electrophoresed and transferred to a PVDF membrane (GE Healthcare, Pittsburgh, PA, USA), which was incubated at 1∶2000 dilution with the primary antibodies. After incubation with horseradish peroxidase-conjugated secondary antibody, expression of each protein was detected using an ECL detection kit (GE Healthcare, Pittsburgh, PA, USA) according to the manufacturer's protocol. The primary antibodies were as follows: rabbit polyclonal antibody against Retnla (the generation of Retnla antibody was previously described); rabbit polyclonal antibody to p38, phosphorylated p38, ERK1/2, phosphorylated ERK1/2, c-Jun N-terminal kinase (JNK), phosphorylated JNK, STAT6, and phosphorylated STAT6 from Cell Signaling Technology (Danvers, MA, USA); and goat polyclonal antibody to GAPDH from Santa Cruz Biotechnology (Dallas, Texas, USA). Band intensities in the immunoblots were quantified by densitometry with TINA 2.0 software.

### Statistical analysis

Results are expressed as the mean ± the standard deviation (SD). Differences between the two animal groups were assessed using a two-tailed non-parametric Mann-Whitney *U*-test. The means of the two groups were considered significantly different if *P*<0.05.

## Results

### Generation of Retnla transgenic mice and experimental allergic asthma model

**Figure 1:**
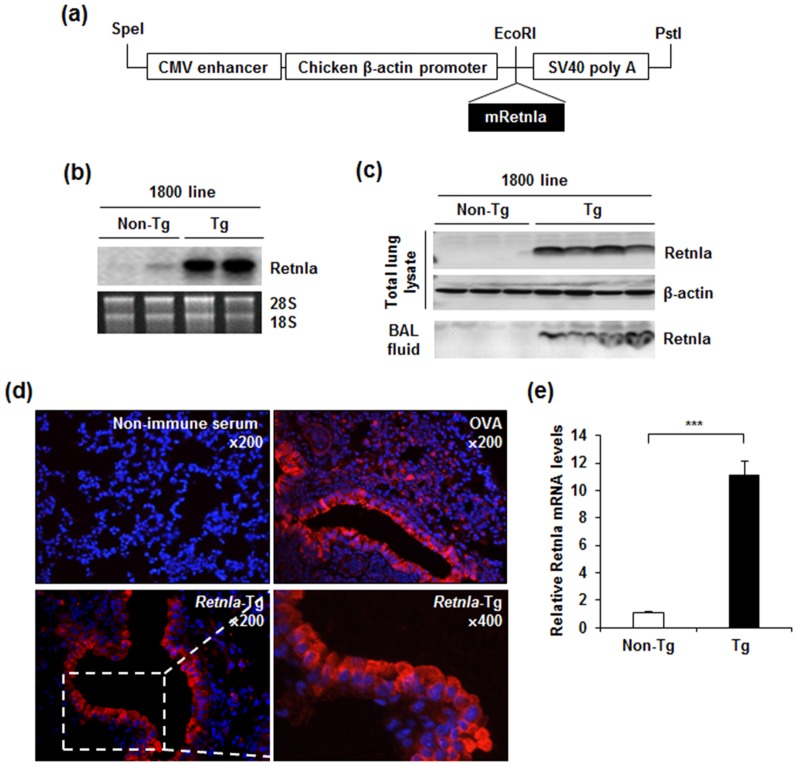
Generation of *Retnla-*Tg mice, and the expression pattern of Retnla in lung. (a) DNA construct for generating *Retnla-*Tg mice. Mouse Retnla cDNA was cloned into the *EcoR*I site of the pCAGGS vector. The transgene was excised as a *Spe*I and *Pst*I fragment for microinjection. (b) Northern blot analysis of total RNA extracted from lungs of *Retnla-*Tg and non-Tg mice. 28S and 18S rRNA were used as controls for RNA amount and integrity. (c) Immunoblot analysis of mouse Retnla from lung and bronchoalveolar lavage (BAL) fluid of *Retnla-*Tg and non-Tg mice. β-actin was also analyzed as a loading control. (d) Control staining using non-immunized rabbit serum. Representative Retnla staining in lungs of OVA sensitized/challenged mice (upper right) and *Retnla-*Tg mice (bottom row). Nuclei were stained with DAPI (blue). Inset: original magnification, 200×. (e) Quantitative RT-PCR analysis showing mRNA expression of Retnla in peritoneal macrophages of non-Tg and *Retnla*-Tg mice (n = 5 per group). Data are represented as mean ±SD. Mann-Whitney U-test; ***P<0.005.

To understand the role of Retnla in a chronic experimental model of allergic asthma, we generated *Retnla*-Tg mice that express Retnla under the control of the chicken β-actin promoter and cytomegalovirus enhancer (Fig. 1a). Two different transgenic (Tg) lines were generated (lines 1800 and 0011). The expression levels of Retnla mRNA and protein in lung tissue were not significantly different between the two Tg lines (Figure S1), both lines were normal in appearance, and there were no histological alterations in parenchymal organs including lung, liver, intestine, and so on. Thus, line 1800 was used in further studies. Retnla mRNA expression was dramatically increased in the lungs of *Retnla*-Tg mice compared with those of non-Tg mice (Fig. 1b). The expression levels of Retnla mRNA in various organs, including skin, brain, heart, kidney, liver, intestine, and adipose tissue, were also dramatically increased in *Retnla*-Tg mice compared with those of non-Tg mice (data not shown). To determine whether the level of Retnla protein in the lung was increased in *Retnla*-Tg mice, Retnla immunoblotting of total lung lysate was performed. *Retnla-*Tg mice had dramatically increased Retnla protein levels than non-Tg mice (Fig. 1c). Since Retnla is known to be a secreted protein [3], we investigated the protein level of Retnla in BAL fluid to examine whether the secretion of Retnla into BAL fluid is also increased in *Retnla*-Tg mice. The protein level of Retnla in BAL fluid was significantly increased in *Retnla*-Tg mice compared to non-Tg mice (Fig. 1c). To investigate the expression pattern of Retnla in lungs of *Retnla*-Tg mice, we performed immunohistochemical staining on lungs from *Retnla-*Tg mice and ovalbumin (OVA)-induced asthmatic mice. Consistent with a previous study [3], the expression of Retnla was mainly localized to bronchial epithelium in OVA-induced asthmatic mice. Although the chicken β-actin promoter, a well-known promoter for overall expression, was used to drive Retnla expression in *Retnla-*Tg mice, the Retnla expression was mostly restricted to bronchial epithelial cells, which is very similar to the pattern in OVA-induced asthmatic lungs (Fig. 1d). In addition, as it is known that macrophages can express Retnla [3], [28], we tested whether macrophage Retnla levels was increased in *Retnla*-Tg mice and observed that the macrophages from *Retnla-*Tg mice had higher levels of Retnla expression than that from non-Tg mice (Fig. 1e), indicating that macrophages may be another major source of secreted Retnla in lung.

### Retnla overexpression did not alter lung histology, respiratory function, or cellular composition under normal conditions

**Figure 2:**
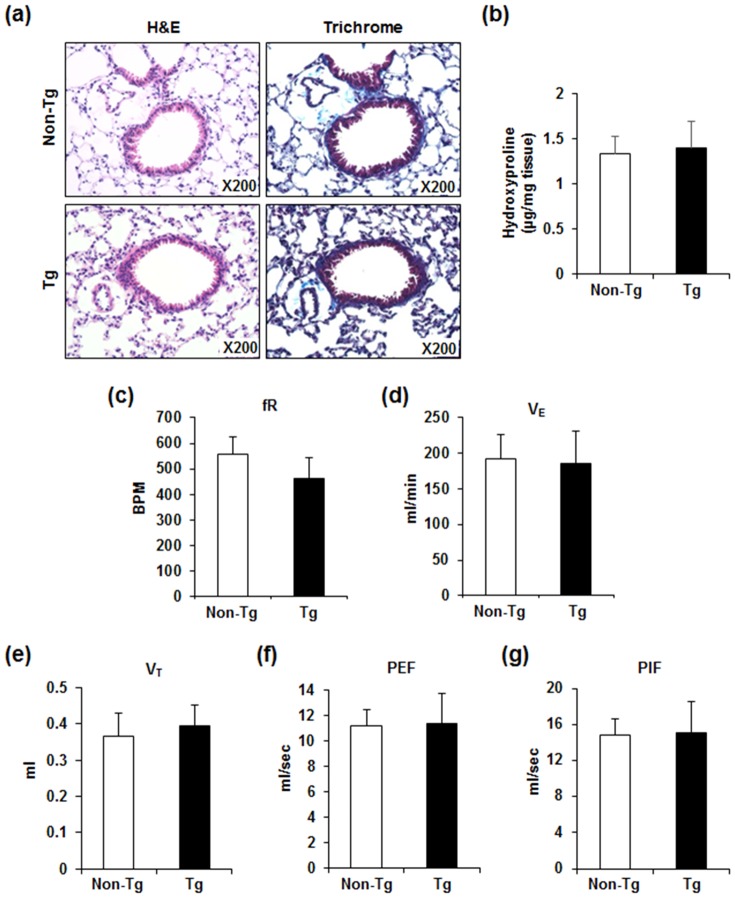
Retnla overexpression did not alter lung histology or function under normal conditions. (a) Representative lung sections from non-Tg (top row) and *Retnla*-Tg (bottom row) mice stained with hematoxylin and eosin (left panel) or trichrome (right panel) (collagen is stained blue). Inset: original magnification, 200×. There was no significant inflammatory change in hematoxylin and eosin staining, and collagen content (blue in trichrome staining) was not changed in *Retnla*-Tg mice. (b) Fibrosis was assessed by the measurement of hydroxyproline in the lungs from non-Tg and *Retnla*-Tg mice (n = 8 for non-Tg, n = 5 for *Retnla*-Tg). (c–g) Analysis of pulmonary functional parameters including (c) respiratory frequency (fR, BPM indicates breaths per minute), (d) minute ventilation (V_E_), (e) tidal volume (V_T_), (f) peak expiratory flow (PEF), and (g) peak inspiratory flow (PIF) were measured using whole-body plethysmography (n = 7 to 8 per group). There were no significant changes in pulmonary function of *Retnla*-Tg mice compared to those of non-Tg mice. Data are presented as mean ±SD. Statistical analysis was performed with the Mann-Whitney *U*-test.

**Figure 3:**
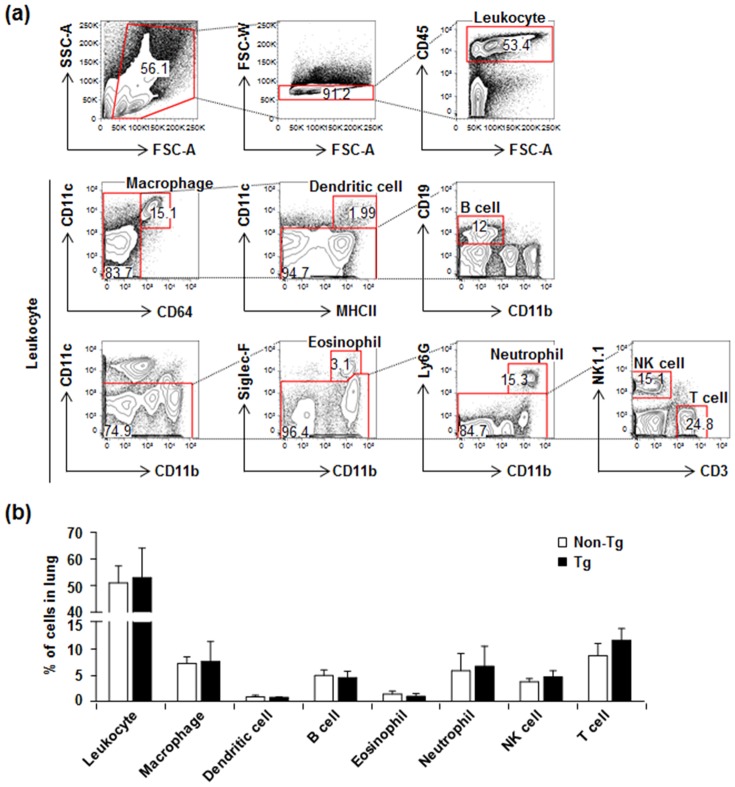
Retnla overexpression did not influence immune cell composition of lung under normal conditions. (a) Gating strategy for identification of immune cell populations from lung of Retnla-Tg and non-Tg mice. (b) Immune cell populations in lungs from *Retnla*-Tg and non-Tg mice were quantified by flow cytometry (n = 7 to 8 per group). Data are presented as mean ±SD. Statistical analysis was performed using the Mann-Whitney *U*-test.

Histological examination showed that Retnla overexpression did not induce histological alterations including airway thickening, inflammatory cell infiltration, or fibrosis in normal lung conditions (Fig. 2a, left panel). Since two conflicting results have been reported regarding the effect of Retnla on lung fibrosis [20], [29], we evaluated whether Retnla overexpression influenced lung fibrosis using Masson's trichrome staining and measured lung hydroxyproline content as an index of tissue collagen accumulation. Consistent with previous observations using lung epithelium-specific *Retnla*-overexpressing mice [29], Retnla overexpression did not induce lung fibrosis under normal conditions in our study (Fig. 2a, right panel and Fig. 2b). We next examined the effect of Retnla overexpression on respiratory responses in normal conditions using whole-body plethysmography to record the breathing of unanesthetized, freely moving *Retnla-*Tg and non-Tg mice. Respiratory frequency (fR), minute ventilation (V_E_), tidal volume (V_T_), peak expiratory flow (PEF), and peak inspiratory flow (PIF) did not differ between *Retnla-*Tg and non-Tg mice (Figs. 2c–g). In histological examination, we did not observe any histopathological changes in the other tissues of *Retnla-*Tg mice. The composition of leukocytes in the normal lungs of *Retnla*-Tg mice was also similar to that of non-Tg mice (Figs. 3a,b).

### Retnla overexpression decreased the immune cell populations of BAL fluid in OVA-induced allergic lung inflammation

**Figure 4:**
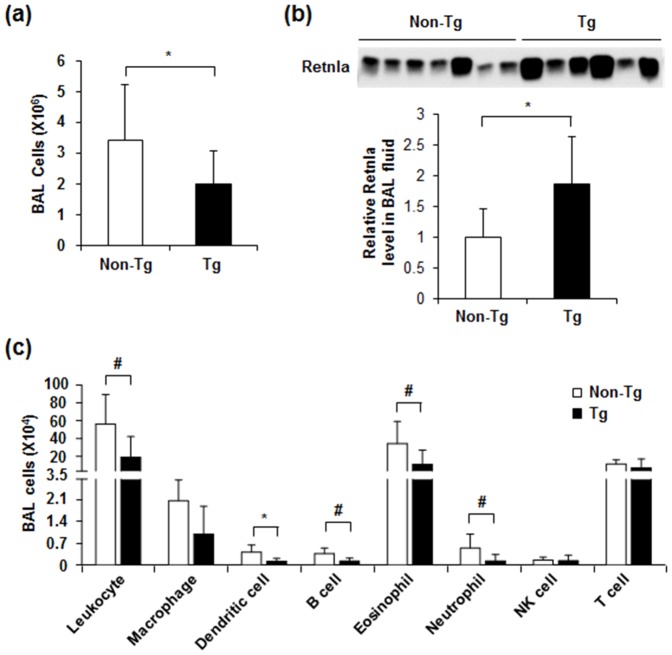
Retnla overexpression reduced immune cells in BAL fluid from OVA-sensitized/challenged mice. (a) Inflammatory cell infiltration was examined in BAL fluid from OVA-challenged *Retnla-*Tg and non-Tg mice (n = 7 to 8 per group). (b) The level of Retnla protein in BAL fluid was analyzed by immunoblotting. Each lane represents an individual mouse. Band intensities were quantitated by computerized densitometry. (c) Immune cell populations in BAL fluid of *Retnla*-Tg and non-Tg mice were quantified by flow cytometry (n = 4 for non-Tg, n = 6 for *Retnla*-Tg). Data are presented as mean ±SD. Statistical analysis was performed with the Mann-Whitney *U*-test; *P<0.05; # *P* = 0.06.

To assess the effect of Retnla overexpression on OVA-induced allergic inflammation, we induced allergic lung inflammation in *Retnla-*Tg mice and non-Tg mice via OVA sensitization/challenge. We found that total number of BAL cells was significantly decreased in *Retnla-*Tg mice compared to non-Tg mice (Fig. 4a). We also observed that Retnla protein level was increased in BAL fluid of *Retnla-*Tg mice after OVA challenge compared to that of non-Tg mice (Fig. 4b). Interestingly, the number of dendritic cells in BAL fluid was significantly decreased in *Retnla*-Tg mice compared with non-Tg mice. Other immune cell populations including macrophages, B cells, eosinophils, and neutrophils were also decreased in BAL fluid in *Retnla*-Tg mice compared with that of non-Tg mice (Fig. 4c).

### Retnla overexpression decreased infiltration of immune cells into the lung tissues and mucus production with no effect on lung fibrosis in OVA-induced allergic lung inflammation

**Figure 5:**
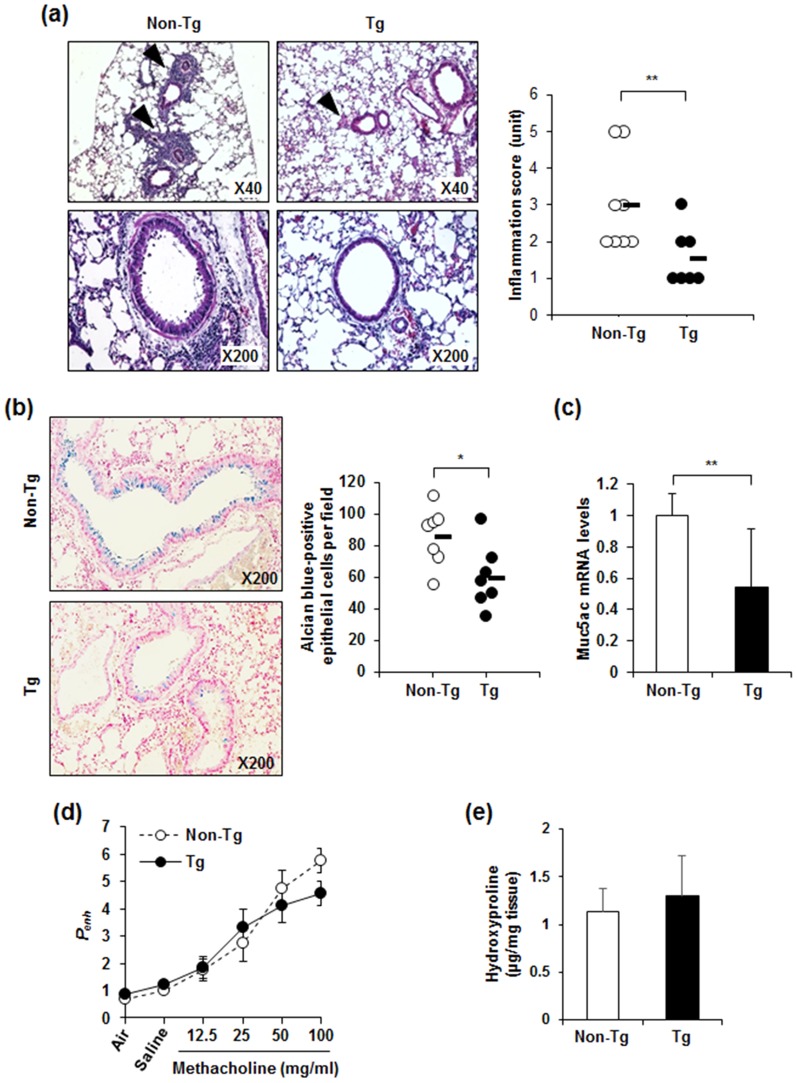
Retnla overexpression attenuated pulmonary inflammation and mucus production in OVA-sensitized/challenged mice. (a) Representative H&E-stained tissue sections demonstrating peribronchial inflammatory infiltrates (arrowheads) in lung. The average histological inflammation score was significantly decreased in *Retnla-*Tg mice. Each dot represents an individual mouse. (b) Representative Alcian blue-stained sections demonstrating mucus production (mucus is stained blue) are shown on the left. The numbers of mucus-producing bronchial epithelial cells per mouse are shown in right. Each dot represents an average number of Alcian blue-positive bronchial epithelial cells from each individual mouse (five sections analyzed/animal, per 200× field). Inset: original magnification, 200×. (c) The mRNA level of Muc5ac was determined by qPCR analysis of RNA from lungs. (d) Airway responsiveness measured in *Retnla-*Tg and non-Tg mice after stimulation with increasing doses of inhaled methacholine (n = 6 per group). (e) Fibrosis was measured by quantifying hydroxyproline level in the lungs. Data are presented as mean ±SD. Statistical analysis was performed using the Mann-Whitney *U*-test; *P<0.05; **P<0.01.

Histopathological examination of the lung sections showed that Retnla overexpression decreased the pulmonary inflammation induced by OVA sensitization/challenge (Fig. 5a). Given that goblet cells produce mucus to protect the airways [30], we performed Alcian Blue staining to evaluate the effects of Retnla overexpression on goblet cell metaplasia and mucus production. Retnla overexpression also significantly reduced the number of mucus-producing epithelial cells within the bronchioles (Fig. 5b). Consistent with this result, we also found that the mRNA level of Muc5ac, which has a pivotal role in mucus production [30], decreased markedly in lungs of OVA-challenged *Retnla*-Tg mice compared to those of OVA-challenged non-Tg mice (Fig. 5c). We next examined airway hyperresponsiveness to inhaled methacholine. The airway hyperresponsiveness was decreased in *Retnla-*Tg mice compared to non-Tg mice in response to the highest dose (100 mg/ml) of methacholine, but the difference was not statistically significant (Fig. 5d). In addition, the presence of lung fibrosis after OVA challenge was not different between the two genotypes (Fig. 5e).

### Retnla overexpression reduced Th2 cytokine production in OVA-induced allergic lung inflammation

**Figure 6:**
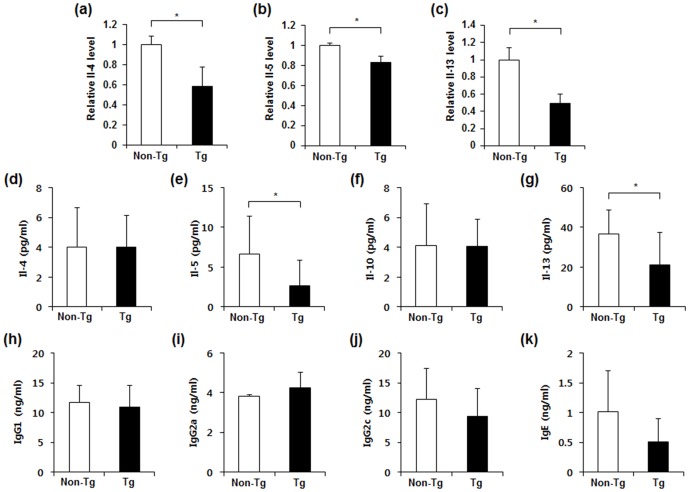
Retnla overexpression decreased the levels of IL-4, IL-5, and IL-13 in OVA-induced asthmatic lung. (a–c) The mRNA levels of IL-4 (a), IL-5 (b), and IL-13 (c) were determined by qPCR analysis of RNA from lungs of OVA-induced *Retnla-*Tg and non-Tg mice (n = 7 to 8 per group). (d–k) Levels of IL-4 (d), IL-5 (e), IL-10 (f), IL-13 (g), IgG1 (h), IgG2a (i), IgG2c (j) and IgE (k) in the BAL fluid were measured by ELISA. Data are presented as mean ±SD. Statistical analysis was performed with the Mann-Whitney *U*-test; *P<0.05.

Th2 cytokines including IL-4, IL-5, and IL-13 have been shown to play crucial roles in the regulation of pulmonary inflammatory responses in allergic asthma [1], [2]. To determine whether Retnla overexpression regulated levels of Th2 cytokines in OVA-induced asthmatic lungs, we measured the lung mRNA levels and BAL protein levels of these cytokines. As shown in Figures 6a-c, *Retnla-*Tg mice showed significant decreases in IL-4, IL-5, and IL-13 in the lung compared to those in non-Tg controls. Similarly, IL-5 and IL-13 levels were markedly decreased in the BAL fluid of *Retnla-*Tg mice compared with that of non-Tg mice (Figs. 6e,g), but IL-4 and IL-10 levels were not decreased in *Retnla-*Tg mice (Figs. 6d,f). In addition, Retnla overexpression did not alter the levels of OVA-specific immunoglobulin (Ig), including IgG1, IgG2a, IgG2c and IgE (Figs. 6h–k). In addition, Retnla overexpression did not affect the M1 or M2 differentiation (Figure S2).

### Retnla overexpression reduced ERK activation in OVA-induced allergic lung inflammation

**Figure 7:**
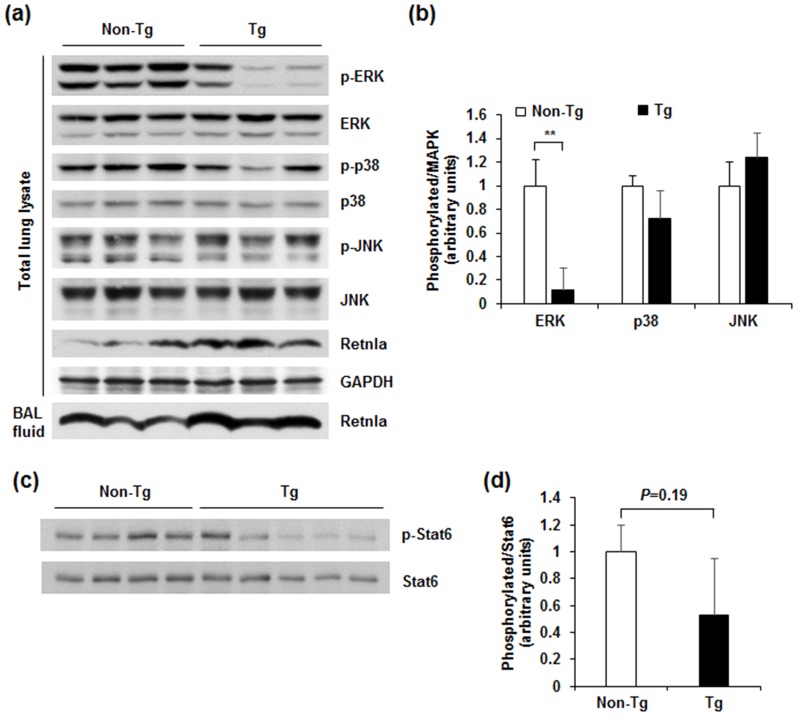
Retnla overexpression reduced ERK activation in OVA-induced asthmatic lung. (a,c) The phosphorylation of MAPKs (ERK, p38, and JNK) (a) and STAT6 (c) in lung tissue was analyzed by immunoblotting. Each lane represents an individual mouse. (b,d) Band intensities in the immunoblot were quantitated by computerized densitometry. The phosphorylation of ERK was markedly reduced in *Retnla*-Tg mice. Data are presented as mean ±SD. Statistical analysis was performed with the Mann-Whitney *U*-test; **P<0.01.

MAPK signaling has an important role in asthmatic lung inflammation [31], [32]. Since asthmatic inflammation was significantly reduced in *Retnla*-Tg mice, we examined the phosphorylation status of ERK, p38, and JNK, which are MAPKs known to mediate proliferative and inflammatory signals induced by inflammatory cytokines in allergic lung inflammation. Figures 7a,b show that *Retnla*-Tg mice have reduced activation of ERK in the lung compared with non-Tg mice, but the phosphorylation of neither p38 nor JNK was significantly different between the groups. The phosphorylation of ERK was also decreased in the lung of *Retnla*-Tg mice under normal condition (Figure S3). Given that the transcription factor STAT6 is critical for regulation of IL-4, IL-5, IL-10, and II-13 and Th2 cell development [33], we tested the effect of Retnla overexpression on STAT6 phosphorylation in lung after OVA challenge. *Retnla*-Tg mice showed a decreasing trend in STAT6 phosphorylation (Figs. 7c,d). Taken together, these results suggest that Retnla attenuates OVA-induced airway inflammation, probably by reducing expression of Th2 cytokines via down-regulation of ERK activity.

## Discussion

In this study, we attempted to determine the exact function of Retnla in asthmatic lung inflammation by generating *Retnla*-overexpressing transgenic mice. Since Retnla can be released into the surrounding environment to affect distant organs, we designed the *Retnla-*Tg vector to be controlled by the chicken β-actin promoter instead of using a tissue-specific promoter. Interestingly, Retnla expression in the transgenic mice was specifically increased in airway epithelial cells and some cells in the alveolar wall, rather than being generally expressed in lung cells. The Retnla expression pattern in the transgenic mice was similar to the situation of allergen-induced lung inflammation. The Retnla protein level was also increased in BAL fluid of the transgenic mice. These results indicate that the final production of Retnla protein may be regulated by a cell type-specific post-translational mechanism that remains to be elucidated.

Retnla has previously been reported to induce fibrosis, thickening of alveolar wall, and epithelial denudation in lung [18], [20]. However, lung epithelial cell-specific Retnla overexpression in mice does not significantly alter fibrosis induced by challenge with bleomycin or silica [29]. Thus, in this study, we attempted to determine whether Retnla overexpression itself could induce histological changes in normal mouse lung. Restrictive lung diseases like lung fibrosis can induce an alteration in several parameters of pulmonary function tests, including tidal volume, peak expiratory flow, peak inspiratory flow, and others [34]. We therefore performed both lung histology and pulmonary functional tests. Retnla overexpression did not induce any alteration in the functional test results compared with those of the non-transgenic controls. Moreover, there were no histological changes, such as fibrosis, epithelial denudation, or thickening of alveolar wall, in *Retnla-*Tg mice. These results indicate that Retnla overexpression does not induce lung fibrosis, which is consistent with the finding in the transgenic mice having specific Retnla overexpression in lung epithelial cells [29].

Several previous papers have reported on the functions of Retnla in inflammation. However, the results of these studies are inconsistent. For example, an animal study using *Retnla*-deficient mice showed that Retnla deficiency exacerbated parasitic infection-induced lung inflammation by enhancing Th2 immune responses, suggesting Retnla to be a negative regulator of Th2 immune responses [14], [15]. In contrast, Retnla deficiency protected against colitis in a dextran sodium sulfate-induced acute colitis model [8], [35]. Moreover, treatment with recombinant Retnla protein has been shown to lead to airway eosinophilia [20], suggesting a proinflammatory property of Retnla. Interestingly, studies using permanent Retnla gene manipulation mostly demonstrated that Retnla prevents lung pathology by decreasing Th2 responses or fibrosis [14], [15], whereas several studies using transient treatment with recombinant Retnla protein or virally-mediated expression of Retnla showed that Retnla exacerbates lung inflammation and fibrosis [18]–[20], [36]. In the present study, we analyzed the effect of Retnla on OVA-induced Th2 type lung inflammation in *Retnla-*Tg mice. Retnla overexpression led to a significant reduction in the number of BAL cells, lung infiltration of inflammatory cells, and mucus secretion into the airway. Moreover, the representative Th2 cytokines IL-4, IL-5 and IL-13 were markedly decreased in *Retnla-*Tg mice compared to the control group. Although there was no statistical significance, the phosphorylation of Stat6, a critical transcription factor for Th2 cytokine induction [33] was decreased in the lungs from *Retnla-*Tg mice. These results indicate that Retnla is a negative regulator of Th2 inflammation, which is in accord with previous studies using *Retnla* knockout mice. In addition, since our results raised the possibility that a decrease in STAT6 signaling may be responsible for the reduction of Th2 cytokines by Retnla overexpression, further studies are needed to understand the exact function of Retnla on the Stat6-mediated Th2 inflammatory processes. Taken collectively, these observations suggest that pulmonary Th2 inflammation may be affected by the expression level and duration of Retnla. This should be clarified by further study.

MAPKs, including ERK, p38 MAPK, and JNK, play pivotal roles in the activation of immune cells [37]. Various stimuli, including cytokines and growth factors, activate MAPK signaling [38], [39]. Among those, ERK activation is often associated with activation of T cells, B cells, and mast cells, leading to proliferation, differentiation, cytokine production, and degranulation [40]–[42]. Recent studies showed that ERK activity was significantly higher in lungs of asthmatic mice than in those of normal controls [43], and that its inhibition had anti-inflammatory effects in OVA-induced mice, leading to reduced production of IL-4, IL-5, and IL-13, as well as decreased airway mucus production and airway hyperresponsiveness [31]. Previous studies have demonstrated that Retnla increased ERK activity, leading to smooth muscle contraction and prolonged survival of myofibroblasts [19], [44]. Those results suggest that Retnla may increase lung inflammation via ERK activation. However, in this study, the activation of ERK was markedly suppressed in asthmatic *Retnla-*Tg mice, which is in contrast with the previous results. Considering that the previous studies were performed in cell culture systems that excluded the effect of tissue-derived cytokines, the suppressed ERK activity in *Retnla-*Tg mice appears to be mediated by reduced Th2 cytokine levels in the lung. Taken as a whole, these results indicate that Retnla overexpression reduces Th2 cytokine levels, resulting in decreased activity of ERK, leading to a further decrease in Th2 cytokine production. Based on our results, including the finding of reduced production of Th2 cytokines, mucus, and ERK activity, we conclude that Retnla overexpression attenuates OVA-induced asthmatic lung inflammation in mice.

## Supporting Information

Figure S1 **Comparison of pulmonary Retnla mRNA and protein expression between 1800 and 0011 mouse line.** (a) Northern blot analysis of total RNA extracted from the lungs of *Retnla*-Tg (1800 and 0011 lines) and non-Tg mice. 28S and 18S rRNA was used as controls for RNA amount and integrity. (b) Representative immunofluorescence staining of Retnla in the lungs from 1800 and 0011 lines. Nuclei were stained with DAPI (blue). Magnification, ×400.
(TIF)

Figure S2 **mRNA expression profiles of M1 and M2-specific markers in peritoneal macrophages.** (a) Quantitative RT-PCR analysis showing mRNA expression of M1 markers in peritoneal macrophages from non-Tg and *Retnla*-Tg mice non-stimulated (control) or stimulated with LPS (100 ng/ml). (b) Quantitative RT-PCR analysis showing mRNA expression of M2 markers in peritoneal macrophages from non-Tg and *Retnla*-Tg mice non-stimulated (control) or stimulated with IL-4 (10 ng/ml). The primer sequences designed for the amplification of each gene are shown in previous report (Nat Commun (2014) 5:4410). Data are represented as mean ±SD. Mann-Whitney U-test; *P<0.05; **P<0.01; ***P<0.005.
(TIF)

Figure S3 **Phosphorylation status of ERK in the lung of** ***Retnla*** **-Tg mouse under normal condition.** Band intensities in the immunoblotting were quantitated by computerized densitometry. The phosphorylation of ERK was markedly reduced in *Retnla*-Tg mice. Data are represented as mean ±SD. Statistical analysis was performed using Mann-Whitney U-test.
(TIF)
