# Supplementary figures and images for: Retnla Overexpression Attenuates Allergic Inflammation of the Airway
Source: PLoS One. 2014 Nov 21;9(11):e112666. doi: 10.1371/journal.pone.0112666 (PMC4240542; doi:10.1371/journal.pone.0112666)

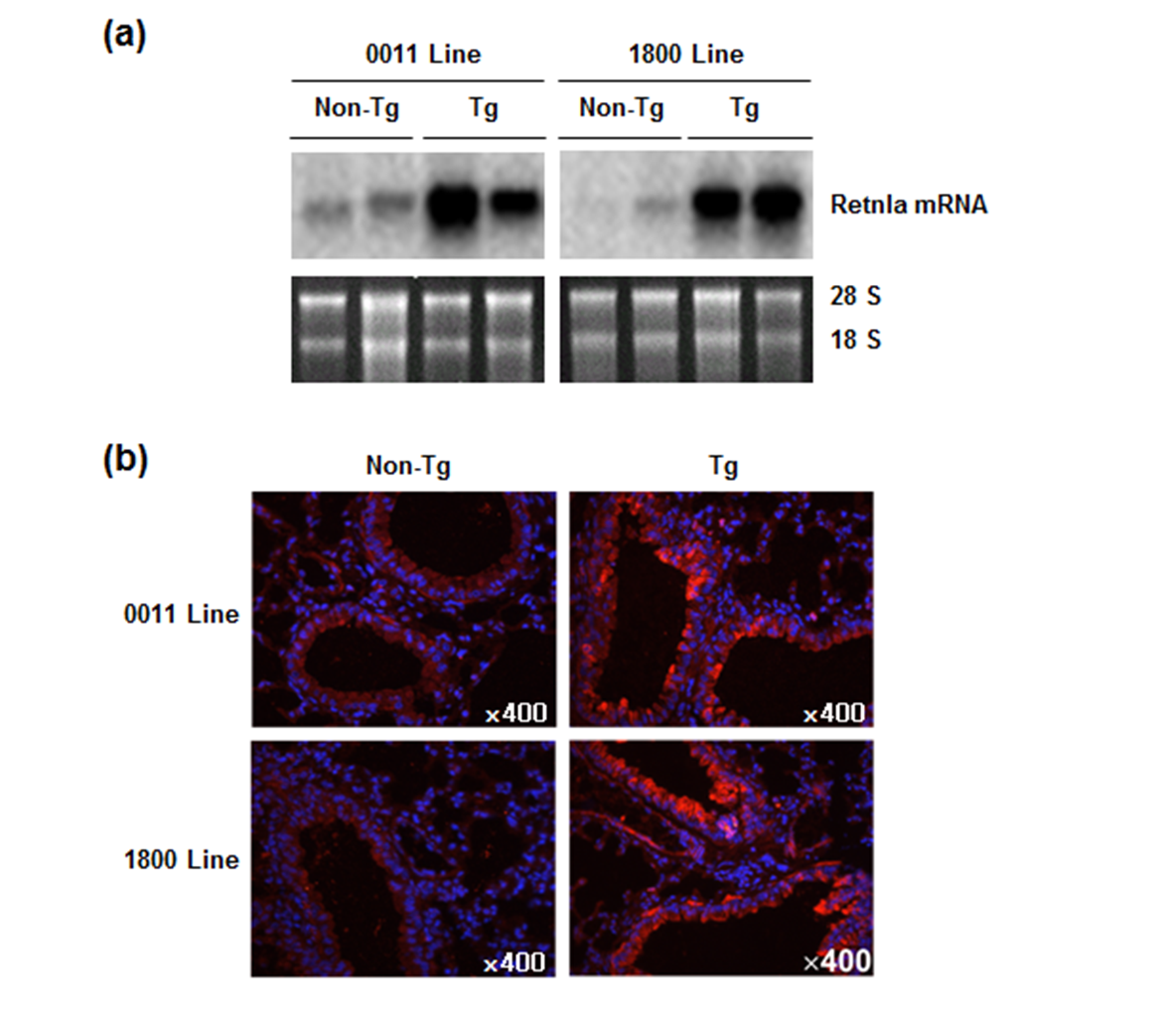

Supplement: Figure S1 — Comparison of pulmonary Retnla mRNA and protein expression between 1800 and 0011 mouse line. (a) Northern blot analysis of total RNA extracted from the lungs of Retnla-Tg (1800 and 0011 lines) and non-Tg mice. 28S and 18S rRNA was used as controls for RNA amount and integrity. (b) Representative immunofluorescence staining of Retnla in the lungs from 1800 and 0011 lines. Nuclei were stained with DAPI (blue). Magnification, ×400. (TIF) [file pone.0112666.s001.tif]

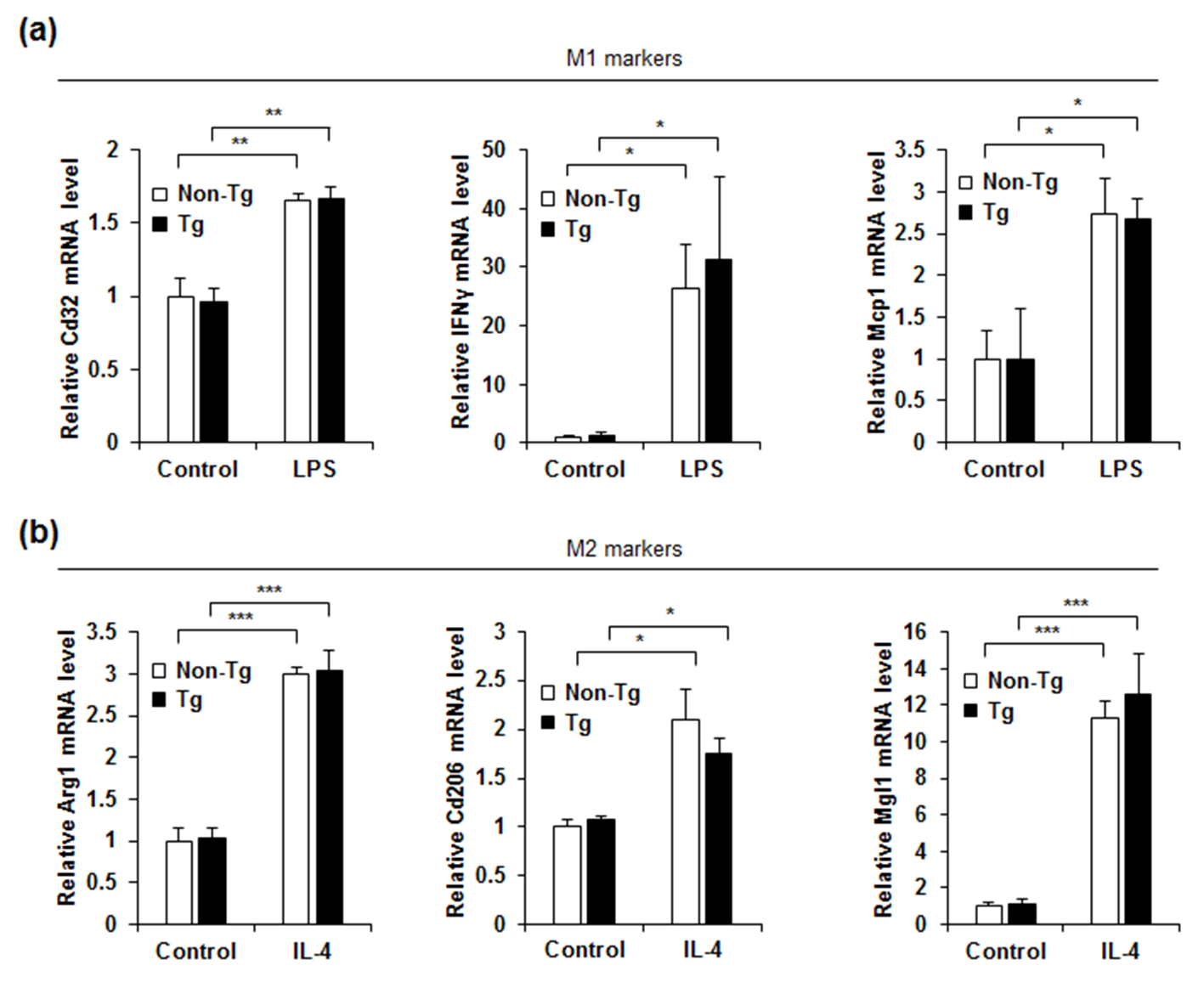

Supplement: Figure S2 — mRNA expression profiles of M1 and M2-specific markers in peritoneal macrophages. (a) Quantitative RT-PCR analysis showing mRNA expression of M1 markers in peritoneal macrophages from non-Tg and Retnla-Tg mice non-stimulated (control) or stimulated with LPS (100 ng/ml). (b) Quantitative RT-PCR analysis showing mRNA expression of M2 markers in peritoneal macrophages from non-Tg and Retnla-Tg mice non-stimulated (control) or stimulated with IL-4 (10 ng/ml). The primer sequences designed for the amplification of each gene are shown in previous report (Nat Commun (2014) 5:4410). Data are represented as mean ±SD. Mann-Whitney U-test; *P<0.05; **P<0.01; ***P<0.005. (TIF) [file pone.0112666.s002.tif]

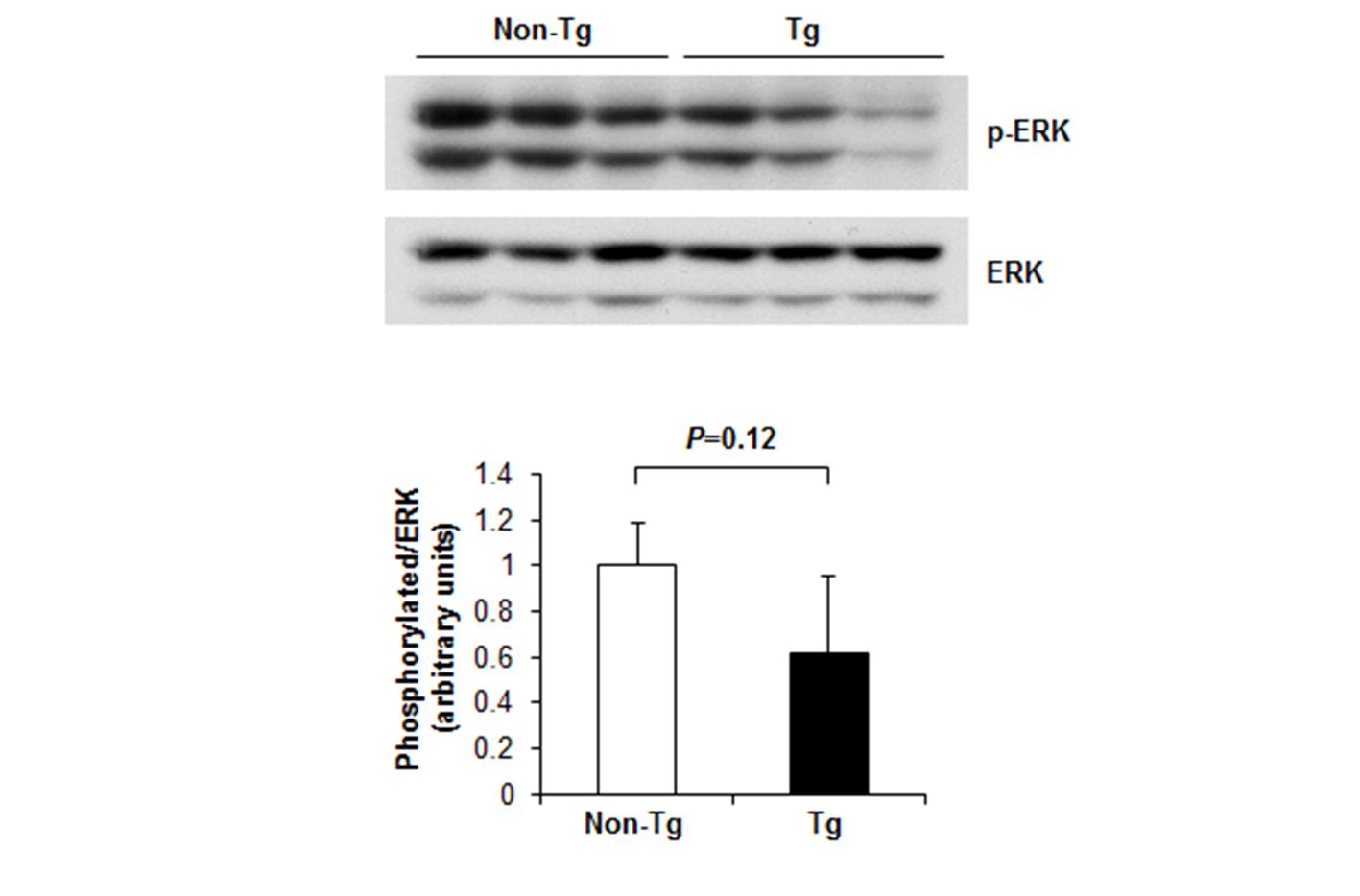

Supplement: Figure S3 — Phosphorylation status of ERK in the lung of Retnla -Tg mouse under normal condition. Band intensities in the immunoblotting were quantitated by computerized densitometry. The phosphorylation of ERK was markedly reduced in Retnla-Tg mice. Data are represented as mean ±SD. Statistical analysis was performed using Mann-Whitney U-test. (TIF) [file pone.0112666.s003.tif]
